# Supplementary material for: Are carbon emissions trading and green financial instruments synergistic? -Comprehensive quantitative research based on content analysis
Source: PLoS One. 2024 Mar 7;19(3):e0298601. doi: 10.1371/journal.pone.0298601 (PMC10919601; doi:10.1371/journal.pone.0298601)
Supplement: S1 Data — (ZIP) [file pone.0298601.s001.zip › Data/地级市Z_F工作报告.zip!/╔╜╢1⁄2/▒⌡╓▌╩╨/└┤╫╘╣1⁄2╓┌║┼í╛┬φ┐╦╩2╛▌═°í┐_2015.docx]

各位代表:

现在，我代表市人民政府向大会报告工作，请予审议，并请市政协各位委员提出意见。

一、关于2014年的工作

在省委、省政府和中共滨州市委的领导下，我们紧紧依靠全市人民，负重奋进，真抓实干，经济和各项社会事业都取得了新成绩。

（一）经济运行实现稳中有进。地区生产总值2277亿元，增长7.6%；一般公共预算收入187亿元，增长10%，税收占比76%，提高0.6个百分点；固定资产投资完成1749亿元，增长15.3%；规模以上工业增加值增长9.6%；社会消费品零售总额739亿元，增长12.2%；金融机构本外币存贷款余额分别达到2106亿元、1870亿元，增长9.3%、10.5%。

（二）结构调整取得积极进展。三次产业结构调整为9.7：50.3：40.0，服务业增加值增长7.2%，占地区生产总值的比重提高1.1个百分点。积极应对严重旱情，粮食喜获丰收，总产达306万吨，增长14.9%。突出科技引领，“渤海粮仓”示范区发展到15.5万亩，黄河三角洲（滨州）国家农业科技园区建设取得新进展，沿黄现代农业示范区成功创建省级示范区。农机深松耕作和机采棉示范区进一步扩大。农业基础建设持续加强，林水会战新增造林19万亩，完成水利工程512项。种养结构不断优化，农林牧渔业总产值430亿元，增长4.4%。工业转调步伐加快，技改投入585亿元，增长18%，预计高新技术产业产值占比达到25.9%，争创省级创新平台22个。积极化解过剩产能，钢铁、电解铝产能列入工信部符合行业规范条件名单。加快现代服务业发展，新增国家4A级景区4个、3A级景区4个，新增省级旅游强镇5个。电子商务发展迅速，在全省率先发展“中国淘宝村”6个。

（三）重点工程项目顺利推进。把滨州港建设作为追赶超越、提质增效的动力引擎，举全市之力加快推进，17公里的西防波堤主体完工，具备开挖深水航道条件，园区内道路等基础设施建设加快，共完成投资17亿元，相当于过去十年全部投资的三分之一。与青岛港集团实现合作，为今年正式开航奠定了坚实基础。加快铁路、高速公路建设，德大铁路滨州段线路建设基本完成，黄大铁路滨州段完成施工招标，济滨东高速滨州段建设进度全线领先、完成总投资的三分之一，秦滨高速埕口至沾化段、长深高速高青至广饶段前期工作顺利推进。鲁中运达保税物流中心通过国家部委联合验收、开关运营。全年5000万元以上固定资产投资项目达到1189个，其中过10亿元的36个，7个项目列入省重点。

（四）城乡建设取得新成效。中心城区路网水系不断完善，“三桥十一路”实现通车，新立河风景带提升工程一期建成，秦皇河北延工程一期完工。抓好市民活动场所建设，“一馆三中心”、市民文化中心部分交付使用，市民体育健身广场建设顺利推进。备受社会关注的中海航母成功招商，国际会展中心实现市场化运营。抓好生态环境建设，秦台河治理完成一期工程，水质明显改善；生活垃圾焚烧发电项目投入运营，市污水处理厂3万吨扩建工程试运行，8个乡镇（街道）获得“国家级生态乡镇”称号。新型城镇建设步伐加快，启动省、市级示范镇1000万元以上项目68个，完成投资36亿元，12个镇成功申报全国重点镇，10个省级示范镇通过省政府考核。农村环境综合整治成效明显，投资22亿元，完成道路硬化2396个村、6354公里，完成净化1832个村、美化2403个村，为建国以来从未修柏油路的69个村全部新修了村庄道路。完成农村电网2128个村变压器、4060公里线路改造。

（五）改革开放有了新的突破。沾化撤县设区，市城区实现沿海发展。市级政府机构改革顺利完成，县级政府机构改革稳步实施，其他领域改革全面推进。创新财政投资模式，争取上级财政投资1亿元参股我市创投基金。组建环海管道燃气输配气公司，“气化滨州”取得新进展。积极推进城乡建设用地增减挂钩、未利用地开发、工矿废弃地复垦，盘活存量土地1.1万亩，供地1.8万亩，确权用海8.5万亩。农村综合产权改革有序推进，农村土地承包经营权确权登记颁证全面推开。大幅精简行政审批事项，市直保留行政许可229项、非行政许可21项。抢抓京津冀一体化机遇，加快推进对外合作，注重招大引强、招才引智，北汽集团、中信集团等4家世界500强企业落地开工，新增国家级引智示范基地1家，省级达到8家。实现进出口总额72.2亿美元，其中出口37.9亿美元，增长6.9%；进口34.3亿美元，下降28.2%。实际到账外资3.8亿美元，增长25.8%。3家企业在“新三板”挂牌，5家上市公司再融资102亿元，滨城区、博兴县各10亿元城投债获批发行，首届资本对接大会信贷签约1021亿元，金融风险防控取得积极成效。滨州港一类开放口岸申报有了实质性进展。

（六）社会民生事业持续改善。始终把增进民生福祉作为政府工作的落脚点，公共财政用于民生支出164.5亿元，占财政支出的61%。承诺的10件民生实事全部完成。新增城镇就业5万人，农村劳动力转移就业10.5万人。城市居民人均可支配收入和农民人均纯收入分别实现30870元、12691元，增长8.8%、11.7%，收入比缩小到2.43:1。居民保险制度不断健全，基本养老保险基础养老金标准进一步提高。养老事业快速发展，被确定为全国养老服务业综合改革试点市。市残疾人职业康复中心建成投用，慈善募捐突破亿元大关。全市保障性住房开工建设8306套，加续建共完成9167套。教育事业全面发展，滨州一中上划市级管理，市城区八中、六中、二中建成启用，与省实验中学联合成立滨州实验中学并首批招生，建成农村校舍标准化项目230个，大力发展学前教育，幼儿入园率明显提升，完善校车监管机制，努力保障学生交通安全。医疗卫生事业取得新突破，在全省率先实现乡镇住院“起付线以上全报销”制度全覆盖，3.3万名患者得到实惠，住院次均和个人付费分别下降23%、54%。新建标准化村级卫生室443处，市公共卫生中心全面建成，市人民医院、中心医院晋升三级甲等医院。人口计生工作完成目标任务。文化体育事业加快发展，县乡村三级公共文化设施全面达标，市文化馆荣获全国文化系统先进集体，新建1200个村健身广场，在第23届省运会上夺得63枚金牌，居全省上游。省级文明城市通过复审，“法治滨州”、“平安滨州”建设深入推进，“全国青少年普法教育示范区”全国考核第一，“平安行•你我他”行动再获全省第一。

（七）政府自身建设不断加强。深入开展党的群众路线教育实践活动，严格落实“八项规定”，坚决整治“四风”问题，工作作风明显改进。严格实施“三公”经费公开，大力压减支出。市直机关清退办公用房5.8万平方米，整体调整安置9个单位。76家中介机构和社会组织实施去行政化。认真执行市人大关于加快依法治市进程的决议，市公共资源交易中心获“中国法治政府奖”提名奖。制定出台了《滨州市重大行政决策程序规定》，主动接受人大监督和政协民主监督，虚心听取各民主党派、工商联、无党派人士和人民团体的意见建议，办理人大代表建议91件、政协提案281件，答复率均为100%。狠抓决策事项落实，对《政府工作报告》分解的108项重点工作责任到人、随机督办。受理市长公开电话3万余个，群众诉求渠道进一步畅通。

各位代表，在经济下行压力增大的情况下，我们能取得较好的成绩，这是省委、省政府和市委坚强领导的结果，是市人大、市政协和社会各界大力支持的结果，是全市上下共同努力的结果。在此，我代表市人民政府，向全市人民、各界人士、驻滨部队官兵和公安干警致以崇高的敬意！向关心支持滨州发展的海内外朋友表示衷心的感谢!

在总结成绩的同时，我们也清醒地看到，我市经济社会发展还存在一些不容忽视的问题：一是面对新形势、新任务，解决新困难、新问题的能力水平不高，改革创新意识不强，精神状态、工作作风还存在不适应的地方，执行落实上还有差距。二是转方式调结构步伐不快，生态环境压力大，企业核心竞争力不强，面对经济下行压力，企业发展困难增多。三是财政、金融压力大，财政刚性支出增多，收入增长放缓，历史欠账偿还集中，保支出、保建设、保偿债任务重；有的企业资金链断裂，企业互保贷款比重高，金融风险隐患需要密切关注。四是政府依法行政仍有不足，廉政建设仍需加强，干部违法违纪问题时有发生，营商环境亟待优化等。对存在的问题，要高度重视，采取积极有效措施，努力加以解决。

二、2015年工作安排

今年是全面深化改革的关键之年，也是全面完成“十二五”规划目标的收官之年。综观今年的宏观形势，世界经济增长仍有不确定性，国内经济正经历阶段性调整，经济发展进入了新常态，增长速度正从高速转向中高速，发展方式正从规模速度型粗放增长转向质量效率型集约增长，结构调整正从增量扩能为主转向调整存量、做优增量并行，发展动力正从要素驱动转向效率驱动和创新驱动。我们必须努力适应新常态对政府工作的新要求，始终保持良好的精神状态，坚定发展信心不动摇，切实做到发展要有新思维，工作要有新作为，作风要有新转变，进一步解放思想，科学实干，创造性地落实上级决策部署，全面完成2015年工作任务。

今年政府工作的总体要求是：深入贯彻落实党的十八大和十八届三中、四中全会精神，坚持稳中求进工作总基调，坚持以提高经济发展质量和效益为中心，坚持“六个更加注重”发展理念，努力在加快转调步伐、突破重点区域、统筹城乡发展、深化改革开放、保障改善民生、构建法治政府上实现新突破，全力加快小康社会建设步伐，把生态美丽幸福新滨州建设提升到一个新水平。

今年经济社会发展的主要预期目标是：地区生产总值增长8.5%左右，一般公共预算收入增长9.5%左右，规模以上工业增加值增长9.5%左右，固定资产投资增长15%左右，进出口总额增长8%左右，社会消费品零售总额增长12.5%左右，城镇和农村居民人均可支配收入分别增长9%左右和10%左右。人口自然增长率控制在8.9‰以内，城镇登记失业率控制在3.6%以内，完成国家和省下达的节能减排目标任务。

为实现上述目标，重点做好六个方面的工作：

（一）坚持稳中求进，努力提升经济发展的质量水平。坚定不移抓好第一要务，把产业结构调整作为工作重点，把创新驱动作为主攻方向，推动传统产业向中高端迈进，努力做到调速不减势、量增质更优。

突出技术创新，大力推动工业产业升级。工业是立市之本、兴市之基。滨州现有的有色金属、冶金、油盐化工、纺织、机械制造、粮食加工等产业，都有一批大的龙头企业，产能名列前茅，要率先抓好骨干企业、主导产业的转型升级，发挥重大引领作用，引导区域经济转型升级。现有骨干企业要加大技改投入，瞄准世界500强、央企、高科技企业搞好合资合作，推进强强联合，抓好工业化和信息化的深度融合，抓好先进工艺、设备的引进消化吸收，努力向下游产品延伸，向高端高质高效迈进，努力改造提升存量。立足滨州实际，发挥广阔的陆域、海港、海域优势和大企业优势，主动承接全国全省特别是京津冀、省会城市群产业转移，新上一批高端大项目。积极争取更多项目进入省重点，扎实推进重大项目建设，落实包保责任，协调解决问题，全力加快建设进度，努力扩大优质增量。强化各类开发区主战场地位，完善奖惩机制，加强考核督导，推动提档升级；完善以企业为主体的产学研协同创新机制，建设一批高水平专业公共技术创新和服务平台；做好要素供应，努力保障重点项目用地、用海需求，加快重点电网工程建设。关心企业家队伍建设，帮助解决生产生活中的实际困难，充分发挥好企业家在转型升级中的引领作用。

突出高效生态，加快农业现代化进程。稳定粮食生产，深入推进高产创建，扩大“十统一”服务面积，努力夺取农业丰收。发挥大田及黄河滩区优质土壤、良好生态和近距离运输的优势，围绕对接京津冀、服务省会城市群，抓好“米袋子”、“菜篮子”、“果盘子”工程，发展好设施农业、水肥一体、名优基地、土地流转、规模经营等现代种养、经营模式，抓好新优特农产品开发和绿色放心食品生产，打造粮食、蔬菜、水果、肉类、渔业、苗木的滨州品牌。树好泰裕麦业、广富畜产公司等一批典型，扶持农业龙头企业，完善生产、加工、保鲜、销售一条龙服务体系，做好农超对接，实现滨州的农产品源源不断地进超市、上餐桌，直接进入消费市场。强化农业科技创新，促进成果转化，加快国家农业科技园区建设，扩大“渤海粮仓”示范面积。搞好“林水会战”，强化农业基础，完善灌排渠系，抓好道路、农田林网建设。

突出扩量提质，提升服务业发展层次。围绕发展生活性服务业，下决心引进一批大项目，加快万达、欧尚、苏宁等大品牌落地步伐，实现城区大型服务业设施突破；大力发展文化体育、养老保健、社区家政等服务业态，抓好农贸市场等规划，搞好便民服务。积极发展现代旅游业，重点打造一批城市旅游品牌和乡村旅游新亮点。围绕发展生产性服务业，大力发展金融保险、信息技术、中介评估、研发设计、法律咨询、会计审计等产业，不断拓展新的增长空间。积极发展新型商业模式，大力发展电子商务，总结推广博兴草柳编、老粗布和沾化冬枣等特色产品网上购销经验，加大电商示范市场、示范企业、示范村培养力度，深化大中型企业电子商务应用。

突出环境优化，发展壮大民营经济。全力营造加快发展的浓厚氛围：思想观念上，坚持权利平等、机会平等、规则平等，对公有制经济和非公有制经济一视同仁；服务支持上，抓好商事登记改革，简化注册登记审批程序，搞好便利化服务，减少行政事业性收费，提高办事效率。落实各项优惠政策：在税收减免、金融服务、科技人才、管理培训等方面，对中小企业大力扶持；放宽市场准入，鼓励民营资本进入金融服务、公用事业、基础设施、城市建设等领域。加强对县区民营经济的考核，努力推动数量增长、质量提升，推动个体工商户转成企业、小企业发展成为规模以上企业，规模以上企业进行公司制改造，培植改制规范企业实现上市。净化市场环境，依法维护企业权益，坚决打击强买强卖、强装强卸、欺诈企业等不法行为，及时受理企业投诉，做到有报必查、违法必究，为企业发展创造良好环境。

（二）发挥区域战略优势，推进重大工程建设。抢抓实施“两区一圈”战略的历史性机遇，主动对接融入，加快推进实施，借势借力做好重大基础设施建设。

抓好港口建设。把港口建设作为推进“两区一圈”开发、振兴经济的重中之重，抓好港口正式通航的各项准备工作，深化与青岛港集团的战略合作，理顺滨州港务集团运管体制，确保6月底前完成2×3万吨码头验收，正式开航运营。开工3万吨深水航道开挖工程，确保年内完成，开工建设2×3万吨液体化工码头。加大用海手续申办力度，加快一类开放口岸申报进度，推进套尔河梯级航道综合整治，强化统一规划管理，健全河海联运体系，依托港口物流，把北部沿海打造成全市产业高地、新的经济增长极。

抓好铁路建设。加快德大铁路工程收尾，抓好滨州、阳信站场整体建设，确保年内正式开通。落实滨港铁路二期投资主体，早日启动建设；配合做好黄大铁路开工，积极推进济青高铁项目，逐步形成北接黄骅港，南连胶济铁路，西接京沪铁路，东达烟台、威海的路网格局。

抓好公路建设。加快济滨东高速公路建设进度，年内完成路基和大中桥工程；争取开工建设长深高速高青至广饶段，尽快实现“县县通高速”的目标；开工建设秦滨高速埕口至沾化段，同时抓好高速公路连接线等配套建设。加快滨惠大道建设进度，抓好大济路、滨温路等国省道维修改建，加强公路养护管理，开展“畅安舒美山东路”创建活动。启动农村公路骨干路网升级改造。全面完成农村道路“三通”工程，完成剩余961个村、2500余公里村庄道路建设。

抓好“十三五”规划。围绕“两区一圈”开发、京津冀一体化区域战略，认真研究谋划好重大基础设施建设，积极做好对上、向外对接，努力争取更多重大工程、重点项目纳入国家和省规划盘子。重点做好铁路、港口、公路、城市交通等专项规划，做好综合交通发展规划，打造现代综合交通枢纽。

（三）围绕宜居宜业，提升城乡建设水平。坚持因势利导、以人为本，构建特色鲜明、布局合理、功能完善的城乡一体发展格局。

建立统一的规划体系。加快《城市总体规划》修编，做好沾化设区等各方面因素衔接，形成涵盖滨城区、沾化区、经济开发区和高新区的一体空间布局。出台《市城区幸福社区规划》，建设一批教育、医疗、银行、农贸市场等便民设施，建成100家社区便民超市，打造市民生活10分钟服务圈。编制好《新型城镇化发展规划》，做好土地利用总体规划调整，推进经济社会发展规划、城乡规划、土地利用规划“三规合一”改革试点。完善村庄规划，尊重历史和传统文化、乡土和自然风貌，优化城乡发展布局。

加强城市建设管理。加快城市基础设施建设，实施市城区东北部供热管网为主的7项集中供热工程，完成秦台河二期治理，启动新立河一期治污工程，抓好北大沟环境治理。加快“气化滨州”建设，增加生产生活用气供应。完善城区道路水系，启动改造提升南环路和西环路，新建改造城区内5条道路，搞好彩虹湖引水和桥梁改造工程，加快白鹭湖、北海公园建设，启动秦皇河滨水新城建设。实施老城区建设管理提升，加大城中村改造力度，抓好集贸市场、道路秩序的综合整治，做好街景和老旧小区供电设施改造。抓好棚户区改造，努力完成82个项目、1.7万户的改造任务。加强城市精细化管理，注重城区设施和环境的检查维修，做好城区道路、公园的绿化美化，抓好城市主干道、出入口、高速路服务区及重要节点的景观建设和卫生环境整治。

建设功能完善的新型城镇。提升各县城市功能，加快推进12个全国重点镇、10个省级示范镇和7个市级示范镇建设，发挥示范带动作用。扎实抓好供水、供电、通信等设施建设。出台户籍改革政策，推进农村转移人口有序转为城镇居民。加大考核力度，确保新型城镇化各项工作落到实处。

打造环境优美的现代乡村。以增减挂钩项目村、城郊村、乡镇驻地村、经济富裕村和大企业、大项目建设需要改造的村为重点，加快农村新型社区建设，逐步将已建成入住的207个农村新型社区纳入城镇化管理。全面完成农村环境综合整治工作，加快推进城乡环卫一体化，引入市场化运营模式，建立完善长效保洁机制。加大农村电网改造力度，完成1990个村变压器、2125公里线路建设任务。

围绕重点工程、城乡建设，研究好融资路子，积极争取发行城投债和政府债券，大力争取棚户区改造资金，采取PPP等多种模式，吸引社会资本参与城乡建设，通过多元化融资，破解资金难题。

（四）加强和改善民生，为群众多办实事。在经济发展新常态下，更要大力改善民生，提高人民群众的幸福指数。

提高社会保障和救助水平。鼓励创业带动就业，全面实施农民工职业技能提升、权益保障、公共服务三项行动计划，拓宽高校毕业生、复退军人、困难群体的就业渠道，实现城镇新增就业3.5万人，农村劳动力转移就业5万人。居民基本养老保险基础养老金标准由每人每月75元提高到85元。研究规范失地农民补偿保障机制。积极应对老龄化，加快养老服务业发展，确保市社会养老服务中心建成投用，开展市社会养老服务中心“公建民营”、敬老院“一院多能”和居家养老服务试点，推进100处养老服务设施建设，新增养老床位3000张。完善社会救助体系，住院大病医疗救助报销比例由60%提高到70%。加大扶贫济困工作力度，实现精准扶贫。

大力发展社会事业。全面提高教育质量，注重内涵发展。启动实施第二期学前教育三年行动计划，改善困难乡镇、薄弱学校教学条件，探索解决小学生校外、留守儿童的社会教育、管理问题，加强校园及周边的安全管理，全面落实校车安全管理机制，推进教育均衡发展。加快发展现代职业教育，启动“职业院校服务产业重点专业建设工程”，支持高等教育办出特色。提高基层医疗水平，抓好乡镇卫生院标准化建设，年底前所有公办基层医疗卫生机构达到省级标准。加快市中医院、妇幼保健院建设进度，完成市人民医院、中心医院门诊综合楼主体工程。城乡居民医保财政补助标准从每人每年320元提高到380元，基本公共卫生服务经费标准从人均35元提高到40元。高度重视人口计生工作，努力提高管理、服务水平，全面完成性别比管控等各项目标。扎实推进公共文化服务体系标准化、基层综合文化中心建设，实施公共文化机构法人治理结构改革，全方位、多层次开展公共文化活动，实施精品创作工程，加强文化市场管理，壮大文化产业，推进社会主义核心价值体系建设。抓好体育惠民工程，完成剩余1200个村的健身设施配建，确保市民体育健身广场竣工开放。加快县城至乡镇、乡镇至村居的公交线路建设改造。全力做好火车通车、港口正式通航、全面完成农村道路“三通”等10件事关人民群众切身利益的民生实事。

加强社会治理创新。依托基层综合服务管理平台，建立健全新型城乡社区治理体系，及时解决土地征用、环境保护等群众反映强烈的问题。强化应急管理，提升应急演练、救援、处置规范化水平。加大食品药品安全监管力度，落实安全生产监管责任，加强消防、交通安全、危爆物品管理，坚决防止重大安全事故发生。推进“平安滨州”建设，完善治安防控体系，严防和惩治各类违法犯罪行为，开展全民禁毒行动，维护社会大局持续和谐稳定。

加快经济社会发展，抓好经济转型升级，离不开人才支撑。要抓好引才引智，加大高层次创新人才和高技能人才引进培养力度，健全完善激励机制，让做出创新贡献的人才获得应有回报，形成依靠科技人才，促进经济社会发展进步的浓厚氛围。

（五）强化生态文明建设，努力优化环境质量。

积极推进节能减排和资源节约。运用法治思维和市场机制，实施源头防控，杜绝高能耗、高污染、高排放、低效益项目上马。制定总量减排计划，强化现有污水处理厂运营监管和提标改造，推进热电项目改造，做好脱硫脱硝工程。大力推行居住、公共建筑节能改造，积极发展节能环保产业，大力推广循环经济和清洁生产先进技术，确保完成年度节能减排任务。落实最严格的耕地保护制度，强化国土执法检查，推动土地节约集约利用。依法依规管海用海，合理开发利用海洋资源。

改善大气环境质量。全面实施大气污染防治规划和一期行动计划，按月公布县（区）空气质量排名，对大气环境质量反弹的区域，实施限批制度。加大黄标车淘汰报废力度，综合治理工业废气、城区扬尘、秸秆焚烧和露天烧烤，力争“蓝天白云、繁星闪烁”天数逐步增加。

加强生态工程建设。做好海河迎查工作，重点抓好断面水质稳定达标，确保再次夺冠。积极开展小清河等重点流域生态环境综合治理，抓好城区水系改造，推动水质持续改善。全面开展全国水生态文明城市建设试点工作，确保通过国家卫生城市复审。严格落实渤海海洋生态红线制度，大力实施渔业资源修复工程。

强化环境执法。认真落实新《环保法》，用最严格的法律制度保护生态环境，重拳打击群众关心关注的偷排偷放、非法排放等恶意环境违法行为，对违法者实施按日计罚、查封扣押、行政拘留，对涉嫌犯罪的，一律迅速移送公安机关。加大环保部门与公安、检察机关的联合协调执法力度，对重点区域、流域实行跨县区联防联控。强化企业社会责任，鼓励企业建立良好的环境信用。

（六）深入推进改革开放，增强区域发展活力。根据中央和省部署要求，着力抓好各领域改革，破除发展障碍，用改革释放潜力，以开放促进发展。

深化行政管理体制改革。围绕精简、效能、廉洁、务实，建立健全行政审批事项目录清单、行政权力清单、政府责任清单、市场准入负面清单，规范行政权力运行。做好上级取消、下放行政审批事项的承接落实，梳理好下放事项，能下放县区的，一律下放到位。加快监管方式转变，建立健全事中、事后监管制度，确保行政审批制度改革有序进行。强化公共资源交易中心建设，不断提高监管、服务水平。完善公共行政审批服务中心窗口功能，确保应进必进、进必授权。抓好中介服务改革，引进更多的市外中介组织来滨州注册落地，建立完善“中介超市”，提升服务质量。

深化农村改革。积极推进农村产权制度改革，基本完成农村土地承包经营权确权登记颁证，探索农村土地承包经营权流转、抵押、担保等办法，抓好农村综合产权交易市场试点。启动省级农田水利产权制度综合改革试点，创新水利设施管护机制。规范发展专业合作社、家庭农场等新型经营主体，推动适度规模经营。加快供销社综合改革，逐步构建新型农村合作服务体系。强化惠农政策落实，扩大农业保险覆盖面。成立农业执法支队，完善农业行政执法制度和程序，维护农民合法权益。

深化财政金融体制改革。改革预算管理，精心搞好市级及部门预算编制，严格按预算安排支出，实行预算公开制度，接受社会监督。突出公共财政导向，加快财政资金退出竞争性领域步伐，采取股权投资基金、融资增信、购买服务、政府与社会资本合作等方式支持经济社会发展。深化政府债务管理改革，清理甄别存量债务，做好融资平台剥离，科学确定发债规模，建立规范的“借、用、还”一体化融资机制。深化农村信用社改革，组建滨州农村商业银行。引导金融单位支持实体经济发展，扩大信贷规模和社会融资总量；强化对域内银行考核，增加域内信贷投放、支持政府项目建设、降低利息费用、防范化解金融风险等工作分值权重。高度重视区域金融风险防范，市级逐步到位设立资本金10亿元的融资性担保公司，每个县区也要设立相应的融资性担保机构；整合土地、企业资产、社会资金等资源做好融资性担保、“过桥”资金等工作，帮助企业“破圈解链”；支持引导企业突出主业，减少无效资金投放，采取用股权换资金、用优质资产寻求合作、到齐鲁和蓝海等区域股权交易市场募集资金等办法，扩大直接融资，缓解资金困难。强化诚信经营，表彰诚信单位，打击恶意欠息逃废债行为。

抓好社会事业改革。推进医药卫生和教育体制改革，逐步改革行政管理模式。推进县级公立医院综合改革，鼓励社会资本举办医疗机构。按照上级统一要求，改革机关事业单位工资制度，调整工资标准，优化薪酬结构。实施机关事业单位养老保险制度改革，建立社会统筹与个人账户相结合的基本养老保险制度。同时，统筹推进国有企业改革、统计制度改革等，圆满完成各项改革任务。

提高对外开放水平。围绕对接京津冀、世界500强、央企、大型民营企业，针对不同区域、骨干产业，成立专门招商队伍，有针对性地搞好对接，提高项目引进的数量和质量。发挥主导产业优势，依托港口腹地，引进一批大型临港产业、先进重化工业、循环经济项目。提高利用外资质量，不仅要引进外部资金，更要注重引进市外、国外实体企业，带动域内产业升级。充分利用国际、国内两个市场，鼓励企业走出去，搞好资源开发、科研合作，做好出口组织工作，不断提高出口产品的质量和档次。加快海关特殊监管区域建设，推动鲁中运达保税物流中心健康运营。

三、加强政府自身建设

做好今年各项工作，必须加强政府自身建设，以更加奋发有为的精神状态、更加勤勉务实的作风、更加坚实有力的举措，努力创造实实在在的工作业绩。

（一）推进依法行政。认真贯彻十八届四中全会精神，全面落实中央和省关于依法治国、法治建设等重大战略部署，坚持在法治轨道上开展工作，加快建设职能科学、权责法定、执法严明、公开公正、廉洁高效、守法诚信的法治政府。坚持科学民主依法决策，健全完善重大行政决策公众参与、专家论证、风险评估、合法性审查、集体讨论决定等制度。深化行政执法体制改革，规范行政执法程序和执法行为，围绕群众关心关注的热点问题，加大执法力度，进一步细化、量化执法裁量权。加强行政复议规范化建设，切实保障人民群众合法权益。加强诚信政府建设，切实提升政府公信力。深入推进政务公开，加大政府投资项目、公共服务信息等公开力度，加强互联网政务信息数据服务平台和便民服务平台建设，提高政务公开信息化水平。认真办理建议提案,自觉接受市人大、市政协和社会公众的监督。

（二）提升服务效能。完善政府机构设置和职能配置，严格控制机构编制，确保财政供养人员只减不增。理清政府部门职责边界，落实工作责任。健全行政绩效管理，强化问责机制，坚决治理不作为、慢作为、乱作为和“为官不为”等问题。全面提升政府部门及领导干部依法行政、执行落实能力，进一步提升大局意识和服务水平。

（三）改进工作作风。巩固党的群众路线教育实践活动成果，健全完善反对“四风”的长效机制。大力弘扬狠抓落实的工作作风，干实事、重实效，把主要精力集中到发展经济、改善民生上来。强化决策事项的检查督导，对研究确定的重点工作和重大事项，以钉钉子的精神一抓到底，善始善终，善做善成。坚持以人为本、民生为先，深入基层一线，扎扎实实地为群众办实事、解难事、做好事，以实际行动服务于民、取信于民。

（四）塑造清廉形象。坚持以身作则，以上率下，全面落实“一岗双责”，严格执行“八项规定”，当好党风廉政建设责任制的推动者和示范者。全面规范办公用房、公务接待，年底前完成党政机关公务用车改革。强化财政资金监管，严控“三公”经费支出，切实把艰苦奋斗、厉行节约的要求落实到工作的各个环节。加强行政效能监察和审计监督，始终保持反腐败高压态势，严肃查处各类违法违纪案件，努力营造风清气正、廉洁高效的政务环境。

各位代表，今年政府工作任务繁重、责任重大。让我们紧密团结在以习近平同志为总书记的党中央周围，在市委的坚强领导下，攻坚克难，开拓创新，为全面完成“十二五”规划目标任务、加快建设生态美丽幸福新滨州而努力奋斗！
